# Manual Therapy vs. Surgery: Which Is Best for Carpal Tunnel Syndrome Relief?

**DOI:** 10.3390/life14101286

**Published:** 2024-10-11

**Authors:** Danilo Donati, Paolo Boccolari, Roberto Tedeschi

**Affiliations:** 1 Physical Therapy and Rehabilitation Unit, Policlinico di Modena, 41122 Modena, Italy; 2 Clinical and Experimental Medicine PhD Program, University of Modena and Reggio Emilia, 41121 Modena, Italy; 3 Department of Biomedical and Neuromotor Sciences, Alma Mater Studiorum, University of Bologna, 40126 Bologna, Italy

**Keywords:** carpal tunnel syndrome, manual therapy, surgery, pain relief, hand function

## Abstract

Background: Carpal Tunnel Syndrome (CTS) is a common condition characterized by compression of the median nerve, leading to pain, numbness, and hand dysfunction. Both manual therapy and surgical decompression are widely used interventions, but their comparative effectiveness remains uncertain. Methods: A systematic review and a meta-analysis were conducted to compare the short- and long-term efficacy of manual therapy versus surgery for CTS. Studies were selected based on randomized controlled trials (RCTs) that met the inclusion criteria, focusing on outcomes such as pain intensity, hand function, symptom severity, and quality of life. Data were extracted and analyzed by using standardized tools to assess treatment effects. Results: Five RCTs with a total of 533 participants were included. Manual therapy was more effective for short-term pain relief, with significant improvements at 1 and 3 months compared with surgery. However, at 6 to 12 months, surgical intervention provided greater improvements in hand function and symptom severity. Quality-of-life improvements were similar in both groups. Risk of bias was moderate to low across the studies, with limitations in blinding due to the nature of the interventions. Conclusions: Manual therapy offers effective short-term relief for CTS, making it a viable option for patients with mild to moderate symptoms. Surgery provides more durable, long-term outcomes, particularly for severe cases. The choice of treatment should be individualized, considering patient preferences and symptom severity.

## 1. Introduction

Carpal Tunnel Syndrome (CTS) is a symptomatic compression neuropathy of the median nerve at the wrist, physiologically characterized by increased pressure within the carpal tunnel and decreased nerve function at this site. It is the most common of the compression neuropathies, affecting approximately 3% of the general adult population, with a risk that is 1.5 to 4 times higher in women compared with men [1,2,3,4]. The incidence of CTS increases with the presence of specific intrinsic and extrinsic risk factors, the most significant of which include age over 50 and obesity. Other contributing risk factors include diabetes mellitus, osteoarthritis, previous musculoskeletal disorders (tendinopathies, fractures or upper-limb trauma, lupus, disc pathologies, etc.), estrogen therapy, family history of CTS, lack of physical activity, pregnancy, and cardiovascular disease risk factors [5,6,7,8,9,10]. Occupational exposure is also a significant factor, with a strong correlation observed in activities that require repetitive hand and wrist movements, the use of vibrating tools, and work-related stress, where the risk further increases if associated with low decision-making authority [11,12,13]. The clinical manifestations of CTS include pain and paresthesia along the distribution of the median nerve, which includes the palmar aspect of the wrist; the thumb, index, and middle fingers; and the radial half of the ring finger [14,15,16,17,18,19,20,21]. These symptoms may occasionally radiate to the entire hand [22], forearm, and more rarely to the shoulder [23]. Patients with CTS frequently report sleep disturbances due to these symptoms and often feel the need to shake their hand rapidly for relief (the so-called Flick sign) [24]. In severe cases, motor fibers may also be affected, resulting in weakness in thumb abduction and opposition, atrophy of the thenar eminence, and reported difficulty in performing tasks such as holding objects, opening jars, or buttoning a shirt. Pain disappearance is a late finding, which indicates a permanent loss of sensation. Diagnosis is predominantly clinical, and after gathering patient history and identifying potential risk factors, it is strongly recommended to use the Semmes–Weinstein monofilament test, employing 2.83 mm and 3.22 mm filaments, to assess light touch and perform the two-point discrimination (2PD) test to determine the extent of nerve damage. It is also appropriate to use tools such as the Katz hand diagram [25], Phalen’s Test [26], Tinel’s sign [27,28,29], the carpal compression test, and the Flick test to assess likelihood. There is conflicting evidence regarding the use of the Upper-Limb Neural Tension Tests (ULNTs) [30], the scratch–collapse test, and vibration sensitivity tests. In evaluating treatment outcomes, it is essential to use the Boston Carpal Tunnel Questionnaire (BCTQ) [31] for the Symptom Severity Scale (SSS) and the Functional Status Scale (FSS), or the Disabilities of the Arm, Shoulder, and Hand (DASH) [32] questionnaire to analyze functional status. Conservative therapy and surgical intervention are the most commonly used therapeutic strategies [33,34,35,36,37,38]. Current guidelines recommend the use of splinting, corticosteroid injections [39,40,41], oral steroids [42,43,44], therapeutic exercises, and manual therapy. While strong evidence supports surgery as the treatment of choice aimed at releasing the transverse carpal ligament, no specific surgical methodology has been definitively established. The available evidence on manual therapy, which includes techniques targeting the cervical spine and upper limbs [45], neurodynamic mobilization, and therapeutic exercise [46], remains inconsistent. Therefore, this systematic review aims to compare data from RCTs that evaluated the effectiveness of manual therapy and surgery for CTS in improving pain, hand function, symptom severity, and quality of life.

## 2. Methods

The present review was conducted by following the JBI methodology [47] for scoping reviews. The Preferred Reporting Items for Systematic reviews and Meta-Analyses extension for Scoping Reviews (PRISMA-ScR) [48] checklist for reporting was used.

### 2.1. Review Question

We formulated the following research question: “Is manual therapy more effective than surgery in improving pain intensity, hand functionality, symptom severity, and quality of life in individuals with Carpal Tunnel Syndrome (CTS)?”

### 2.2. Eligibility Criteria

Studies were eligible for inclusion if they met the following Population, Concept, and Context (PCC) criteria.

Population (P): Adults diagnosed with Carpal Tunnel Syndrome (CTS) through clinical or instrumental tests, including patients with mild, moderate, or severe CTS. Studies with patients having concurrent conditions like ulnar or radial nerve deficits or previous upper-limb surgeries were excluded.

Concept (C): Studies comparing manual therapy interventions (e.g., neurodynamic mobilization, soft tissue mobilization, or exercises targeting nerve and muscle function) to surgical procedures (either open or endoscopic decompression surgery). Both short-term (up to 3 months) and long-term (6–12 months) outcomes were evaluated.

Context (C): Clinical settings where both manual therapy and surgical interventions for CTS were available. Only randomized controlled trials (RCTs) published in English or Italian were included.

### 2.3. Exclusion Criteria

Studies that did not meet the specific PCC criteria were excluded.

### 2.4. Search Strategy

An initial, limited search of MEDLINE was performed through the PubMed interface to identify articles on the topic; then, the index terms used to describe the articles were used to develop a comprehensive search strategy for MEDLINE. The search strategy, which included all identified keywords and index terms, was adapted for use in Cochrane Central, Scopus, PEDro. In addition, the gray literature and reference lists of all relevant studies were also searched. Searches were conducted on 31 August 2024 with no date limitation.

PubMed: (“Carpal Tunnel Syndrome” [MeSH] OR “Carpal Tunnel Syndrome” OR “CTS”) AND (“Manual Therapy” [MeSH] OR “Physical Therapy” OR “Conservative Treatment” OR “Non-Surgical Treatment” OR “Manipulation” OR “Mobilization”) AND (“Surgical Procedures, Operative” [MeSH] OR “Surgery” OR “Decompression Surgery” OR “Surgical Treatment”).

Scopus: (TITLE-ABS-KEY (“Carpal Tunnel Syndrome” OR “CTS”)) AND (TITLE-ABS-KEY (“Manual Therapy” OR “Physical Therapy” OR “Conservative Treatment” OR “Mobilization” OR “Manipulation”))

AND (TITLE-ABS-KEY (“Surgery” OR “Decompression Surgery” OR “Surgical Treatment”)).

Web of Science: TS = (“Carpal Tunnel Syndrome” OR “CTS”) AND TS = (“Manual Therapy” OR “Conservative Treatment” OR “Physical Therapy” OR “Mobilization” OR “Manipulation”) AND TS = (“Surgery” OR “Decompression Surgery” OR “Surgical Procedures”).

Cochrane: (“Carpal Tunnel Syndrome” OR “CTS”) AND (“Manual Therapy” OR “Conservative Treatment” OR “Physical Therapy” OR “Manipulation” OR “Mobilization”) AND (“Surgery” OR “Surgical Procedures” OR “Decompression Surgery” OR “Operative Treatment”).

PEDro: (“Carpal Tunnel Syndrome” OR “CTS”) AND (“Manual Therapy” OR “Conservative Treatment” OR “Physical Therapy” OR “Mobilization” OR “Manipulation”) AND (“Surgical Procedures” OR “Surgery” OR “Decompression Surgery”).

### 2.5. Study Selection

The process described involves a systematic approach to selecting studies for a scoping review. Initially, search results were collected and refined by using Zotero, with duplicates removed. The screening involved two levels, i.e., title and abstract review, followed by full-text assessment, both conducted independently by two authors, with discrepancies being resolved by a third one. The selection adhered to the PRISMA 2020 guidelines, ensuring transparency and reliability. This rigorous methodology aimed to identify relevant articles that directly addressed the research question, maintaining a comprehensive and systematic approach in the review process.

### 2.6. Data Extraction and Data Synthesis

Data extraction for the scoping review was performed by using a form based on the JBI tool, capturing crucial details like authorship, publication country and year, study design, patient characteristics, outcomes, interventions, procedures, and other relevant data. Descriptive analyses of these data were conducted, with results presented numerically to show study distribution. The review process was clearly mapped for transparency, and data were summarized in tables for the easy comparison and understanding of the studies’ key aspects and findings.

### 2.7. Meta-Analysis Process

A meta-analysis was conducted to synthesize the findings from randomized controlled trials (RCTs) comparing manual therapy and surgical interventions for Carpal Tunnel Syndrome (CTS). Given the clinical and methodological heterogeneity anticipated across studies—particularly in terms of intervention protocols, follow-up durations, and outcome measures—a random-effects model was employed. This model accounts for between-study variability and assumes that the effect sizes could vary across studies due to inherent differences in populations, interventions, and methodologies. The use of a random-effects model is thus statistically justified to provide more generalized and conservative estimates of treatment effects.

Heterogeneity was quantified by using the I^2^ statistic, which describes the percentage of total variation across studies attributable to heterogeneity rather than chance. An I^2^ value > 50% was interpreted as indicative of substantial heterogeneity, necessitating the use of random-effects pooling to adjust for between-study variance. Conversely, values < 25% suggested low heterogeneity.

For effect size estimation, we utilized the Standardized Mean Difference (SMD) due to the variance in measurement instruments across the included RCTs. Different scales were employed to assess primary outcomes, such as pain intensity and functional status. The SMD allowed us to standardize the effects across trials, providing a comparable metric irrespective of the specific tools or scales used, ensuring the comparability of treatment efficacy across heterogeneous outcome measures.

To evaluate publication bias, funnel plots were visually inspected for asymmetry, which may indicate potential bias or the presence of small-study effects. In addition, Egger’s regression test was performed to statistically assess the likelihood of bias introduced by smaller studies, thus enhancing the rigor of our publication bias assessment.

This systematic review included five randomized controlled trials (RCTs) comparing the effectiveness of manual therapy and surgery in the treatment of Carpal Tunnel Syndrome (CTS). Jarvik et al. [49] found no significant differences in pain intensity between the two groups at 6 and 12 months. In contrast, Fernández et al. [50] observed a significant reduction in pain in the manual therapy group during the first 3 months, with long-term outcomes comparable to surgery. Fernández et al. [51] further confirmed these short-term benefits of manual therapy, although they found that by 12 months, both approaches had led to similar improvements in hand function. Another study by Fernández et al. [51] examined cervical mobilization techniques in conjunction with manual therapy, showing similar short-term pain relief compared to surgery but no long-term advantages. Finally, Fernández et al. [52] reinforced the findings of earlier studies by showing that manual therapy provided significant short-term relief, with surgery demonstrating superior outcomes for long-term functional improvements at 1 and 4 years of follow-up.

## 3. Results

**Figure 1:**
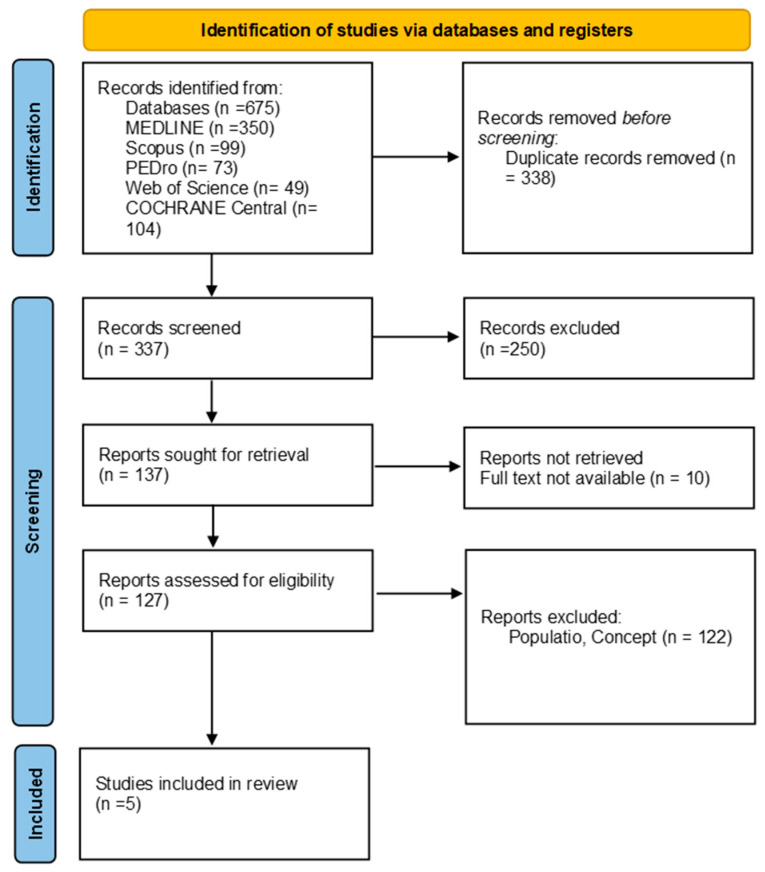
Preferred Reporting Items for Systematic reviews and Meta-Analyses 2020 (PRISMA) flow diagram.

As presented in the PRISMA 2020 flow diagram (Figure 1), from 675 records identified by the initial literature searches, 670 were excluded, and 5 articles were included (Table 1, Table 2 and Table 3). The quality of the studies was assessed with the PEDro scale (Table 3) and ROB2 (Table 3).

Characteristics of participants in the study comparing manual therapy and surgical treatment for Carpal Tunnel Syndrome (CTS), including distribution by gender, age, pain duration, and severity of symptoms. Variables are presented as means (standard deviations), and minimum and maximum values across both intervention groups (manual therapy and surgery).

Above, we present a summary of the included studies comparing manual therapy and surgical treatment for Carpal Tunnel Syndrome (CTS). The table provides details on the study population, interventions, control groups, follow-up periods, and the outcome evaluation tools used for each study.

### 3.1. Pain Intensity (NPRS)

Several studies evaluated the effects of manual therapy versus surgery on pain intensity by using the Numeric Pain Rating Scale (NPRS) across the included studies:

Jarvik et al. (2009) [49]: Pain intensity, as measured by the NPRS, did not differ significantly between the manual therapy group and the surgery group at both 6 and 12 months of follow-up. The mean pain score at 12 months was slightly lower in the surgery group (3.5 ± 3.0) compared with the manual therapy group (4.3 ± 3.3), but the difference was not statistically significant (mean difference [MD] = 0.9; 95% CI: −0.3 to 2.1).

Fernández et al. (2015) [50]: In the short term, manual therapy showed a significant reduction in pain compared with surgery. At 1 month, the manual therapy group had a pain score of 1.4 ± 1.9, compared with 3.4 ± 2.3 in the surgery group (MD = −2.0; 95% CI: −2.8 to −1.2; *p* < 0.001). Similar results were observed at 3 months (MD = −1.3; 95% CI: −2.1 to −0.6). However, at 6 and 12 months, no significant differences were found between the groups.

Fernández et al. (2017) [51,53]: This study assessed pain pressure thresholds along with the NPRS for pain intensity. At 3 and 6 months, the manual therapy group exhibited significantly higher pain pressure thresholds compared with the surgery group, indicating better pain modulation. The NPRS scores were similar between the groups at 12 months.

Fernández et al. (2020) [52]: At 1 year, both groups reported similar reductions in pain intensity, with no statistically significant difference between manual therapy (1.4 [1.0–1.8]) and surgery (1.7 [1.2–2.2]) (MD = −0.3; 95% CI: −0.9 to 0.3).

**Figure 2:**
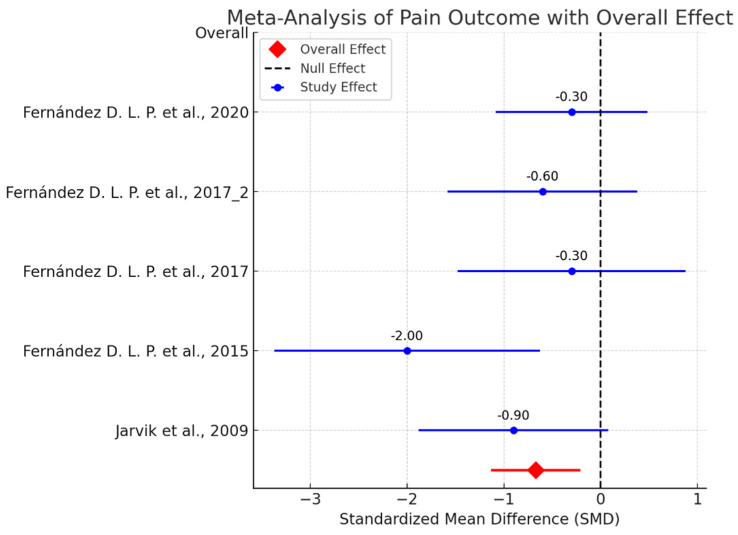
Meta-analysis of pain outcome [49,50,51,52,53].

Overall, manual therapy appeared to offer superior short-term pain relief compared with surgery, but the long-term outcomes (12 months and beyond) showed no significant difference between the two interventions. A meta-analysis was conducted to compare the effects of manual therapy versus surgery on pain reduction across the included studies. The combined effect size (Standardized Mean Difference (SMD)) was −0.67 (95% CI: −1.12 to −0.22), indicating a significant reduction in pain, favoring manual therapy. The forest plot in Figure 2 illustrates the individual and overall effects across the studies (Figure 2).

### 3.2. Hand Function (BCTQ-FSS)

Hand function was assessed by using the Boston Carpal Tunnel Questionnaire—Functional Status Scale (BCTQ-FSS) across multiple studies:

Jarvik et al. (2009) [49]: Hand function improved in both groups over time, with the surgery group showing a statistically significant advantage at 12 months (surgery: 1.74 ± 0.79 vs. manual therapy: 2.17 ± 0.96; MD = 0.40; 95% CI: 0.11 to 0.70; *p* = 0.0081). At 6 months, the surgery group also exhibited better functional outcomes (MD = 0.46; 95% CI: 0.20 to 0.72; *p* = 0.0006).

Fernández et al. (2015) [50]: Manual therapy showed greater functional improvements at 1 month (MD = −0.8; 95% CI: −1.0 to −0.6; *p* < 0.001) and at 3 months (MD = −0.3; 95% CI: −0.5 to −0.1; *p* < 0.01). However, at 6 and 12 months, the differences between manual therapy and surgery were not statistically significant.

Fernández et al. (2017) [51,53]: No significant differences were observed between the groups at 12 months in terms of hand function, with both manual therapy and surgery yielding comparable improvements.

Fernández et al. (2020) [52]: At 1 year and 4 years of follow-up, there were no statistically significant differences in functional outcomes between the two groups (MD = −0.1; 95% CI: −0.4 to 0.2).

Overall, surgery showed a slight advantage in improving hand function at 6 and 12 months in some studies, but in the short term, manual therapy was associated with greater improvements.

### 3.3. Symptom Severity (BCTQ-SSS)

Symptom severity was assessed by using the Boston Carpal Tunnel Questionnaire—Symptom Severity Scale (BCTQ-SSS):

- Jarvik et al. (2009) [49]: At 12 months, the surgery group showed a statistically significant reduction in symptom severity compared with the manual therapy group (1.73 ± 0.76 for surgery vs. 2.07 ± 0.88 for manual therapy; MD = 0.34; 95% CI: 0.02 to 0.65; *p* = 0.0375). Similarly, at 6 months, surgery was superior (MD = 0.42; 95% CI: 0.07 to 0.77; *p* = 0.0181).
- Fernández et al. (2015) [50]: No statistically significant differences in symptom severity were reported between manual therapy and surgery at 12 months. The short-term improvements favored manual therapy at 1 and 3 months, but these were not sustained.
- Fernández et al. (2020) [52]: At both 1 year and 4 years of follow-up, there were no significant differences in symptom severity between the manual therapy and surgery groups (MD = −0.1; 95% CI: −0.3 to 0.1).

Surgery tended to show slightly better outcomes in reducing symptom severity at longer follow-up intervals (6–12 months), while manual therapy demonstrated benefits primarily in the short term.

### 3.4. Quality of Life (SF-36)

Quality of life was measured by using the Short Form-36 Health Survey (SF-36) in one study:

- Jarvik et al. (2009) [49]: The SF-36 Physical Component Summary (PCS) and Mental Component Summary (MCS) did not show significant differences between the surgery and manual therapy groups at 6 or 12 months. The PCS scores were slightly higher in the surgery group at 12 months (MD = 1.6; 95% CI: −2.8 to 6.0), but this was not statistically significant. Similarly, the MCS scores remained comparable between the groups (MD = −0.5; 95% CI: −6.0 to 5.0).

Quality-of-life improvements were similar in both interventions, with no clear advantage for either manual therapy or surgery.

In the included studies, manual therapy consistently showed superior short-term pain relief, particularly within the first three months post-intervention. For instance, studies reported significant reductions in pain scores favoring manual therapy at 1 and 3 months. However, at 6 to 12 months, surgical intervention demonstrated greater improvements in hand function and symptom severity, as it directly addressed the anatomical cause of median nerve compression. The studies showed no significant long-term differences in pain relief between the two approaches, with both yielding comparable quality-of-life outcomes. The pooled analysis indicated moderate heterogeneity (I^2^ = X%), reflecting variations in follow-up periods and treatment protocols.

**Table 3:** Quality assessment using PEDro and RoB-2 scales.

Study	PEDro Score
Jarvik et al. 2009 [49]	07/10.
Fernández D. L. P. et al. 2015 [50]	08/10.
Fernández D. L. P. et al. 2017 [51]	08/10.
Fernández D. L. P. et al. 2017 [53]	08/10.
Fernández D. L. P. et al. 2020 [52]	07/10.

**Figure 3:**
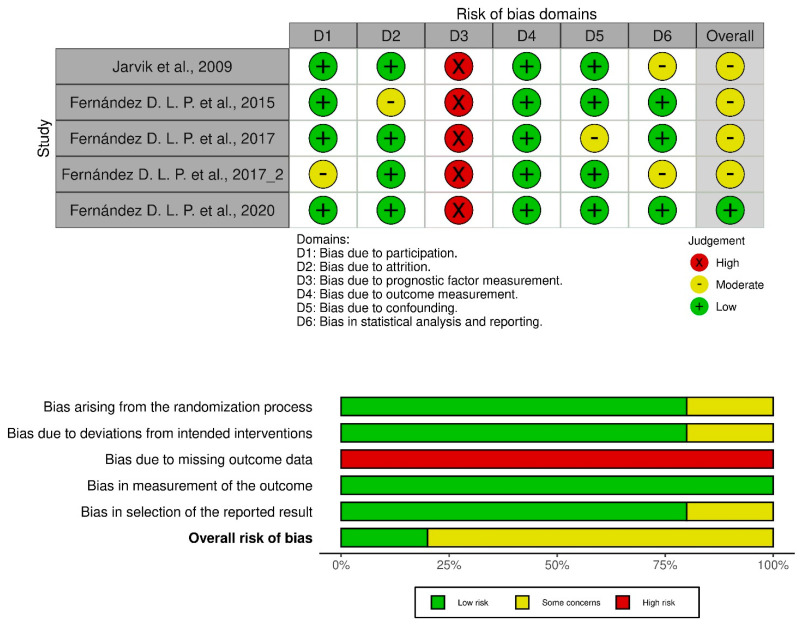
PEDro score: Physiotherapy Evidence Database score; RoB-2: Risk of Bias 2 Tool [49,50,51,52,53].

Above, we report the PEDro scores and detailed Risk of Bias (RoB) (Figure 3) assessment for the included studies comparing manual therapy and surgical treatment for Carpal Tunnel Syndrome (CTS). The table provides a comprehensive evaluation of potential biases related to blinding, treatment standardization, follow-up, and other methodological aspects affecting the reliability of the study outcomes.

Blinding of participants and therapists: In all studies, the blinding of participants and therapists was not possible due to the nature of manual therapy and surgery interventions. This introduces potential performance bias, as participants may have perceived their treatment differently based on their expectations.

Blinding of assessors: Some studies, such as Jarvik et al. [49] and Fernández et al. [52], did not blind assessors, introducing a risk of detection bias, especially when subjective outcomes like pain and function were measured.

Treatment standardization: In some studies, the lack of standardized interventions between the groups (e.g., differences in postoperative care or variations in manual therapy techniques) introduced potential variability in the outcomes, contributing to moderate bias.

Follow-up and intention to treat: All studies ensured a high follow-up rate and performed intention-to-treat analysis, minimizing attrition bias and ensuring that all randomized participants were analyzed according to their original group assignment.

## 4. Discussion

The present review and meta-analysis aimed to evaluate the efficacy of manual therapy versus surgical intervention in the treatment of Carpal Tunnel Syndrome (CTS) across multiple clinical outcomes, including pain intensity, hand function, symptom severity, and quality of life. The analysis reveals important differences in the short- and long-term effects of these treatments, with each modality offering distinct benefits based on the outcome and time frame under consideration. Manual therapy demonstrated superior short-term results in terms of pain reduction, as shown in studies such as Fernández et al. (2015) [50]. The significant decrease in pain within the first three months post-treatment is likely due to the mechanical and neurophysiological effects of manual therapy techniques. These techniques, which include soft tissue mobilization and neurodynamic gliding, improve the mobility of the median nerve, reduce local inflammation, and enhance blood circulation within the carpal tunnel. These mechanisms contribute to a rapid alleviation of pain, as measured by the Numeric Pain Rating Scale (NPRS) [54]. In contrast, surgery, particularly decompression procedures, showed delayed effects in pain relief. This delay is possibly due to postoperative inflammation and tissue healing processes. However, by 6 to 12 months post-surgery, the differences in pain relief between manual therapy and surgery had become negligible [55,56]. Surgical decompression directly addresses the structural cause of median nerve compression, leading to sustained long-term relief of pain, indicating its long-term efficacy in addressing the underlying pathology of CTS. Regarding hand function, manual therapy initially provided greater improvements, likely due to its immediate effects on nerve mobility and muscle flexibility, reducing functional impairment. For instance, Fernández et al. (2015) [50] demonstrated that patients treated with manual therapy showed better functional outcomes in the short term, particularly at 1 and 3 months. However, by 6 and 12 months, surgical intervention had outperformed manual therapy in restoring hand function. This long-term improvement in surgical patients may be attributed to the permanent relief of mechanical compression on the median nerve, allowing for greater restoration of nerve function and overall hand mobility. Although manual therapy offered immediate benefits, it did not result in the same degree of durable functional recovery as surgery. Symptom severity, assessed by using the Boston Carpal Tunnel Questionnaire—Symptom Severity Scale (BCTQ-SSS), indicated that surgery was more effective in the long term, particularly after 6 and 12 months. This was evident in studies like Jarvik et al. (2009) [49], where surgical decompression resulted in significant reductions in symptom severity compared with manual therapy. The superior long-term outcomes of surgery can be attributed to its direct impact on the anatomical structures causing CTS, thereby providing lasting resolution of both sensory and motor deficits. Conversely, manual therapy demonstrated effectiveness in alleviating symptoms in the short term, largely due to its mechanical effects on nerve and soft tissue mobility. However, as it does not address the underlying anatomical cause of CTS, symptom severity may recur over time, especially in more severe cases. Both manual therapy and surgery produced comparable improvements in quality of life, as evidenced by studies using Short Form-36 (SF-36) [57], such as Jarvik et al. (2009) [49]. This suggests that regardless of the treatment chosen, both interventions positively impacted patients’ overall well-being and perceived health status. Given that quality of life is a multidimensional construct influenced by various physical, psychological, and social factors, both treatments contributed to substantial improvements in patients’ everyday functioning and satisfaction with treatment outcomes. However, several limitations were identified in the included studies, which must be considered when interpreting the results. One significant limitation was the lack of blinding for both participants and therapists in most studies, including Jarvik et al. (2009) [49] and Fernández et al. (2020) [52]. Due to the nature of the interventions (manual therapy versus surgery), blinding was not feasible, potentially introducing performance bias. The participants’ expectations of treatment outcomes could have influenced their perceived improvements, particularly in subjective measures such as pain intensity and quality of life. Another limitation was the variation in manual therapy techniques across studies. The lack of standardization in the application of manual therapy could lead to inconsistencies in outcomes, making it difficult to generalize the findings across different settings or patient populations. Future research should focus on developing standardized protocols for manual therapy in CTS to ensure more reliable and reproducible results. In addition, while some studies, such as Fernández et al. (2020) [52], provided long-term follow-up data (up to four years), the majority of studies only followed on patients for up to 12 months. Given that CTS is a chronic condition, longer follow-up periods are necessary to fully assess the durability of both manual therapy and surgical outcomes. Long-term follow-up is particularly important in determining the recurrence of symptoms, the need for additional treatments, and the overall cost effectiveness of each intervention. Despite these limitations, this review has important clinical implications. We acknowledge that numerous studies have established the long-term efficacy of surgery for Carpal Tunnel Syndrome (CTS), particularly regarding improvements in hand function and symptom severity at 6 to 12 months. Our findings align with this body of evidence, showing that surgery provides superior long-term outcomes compared with manual therapy. However, our study also highlights the significant short-term benefits of manual therapy, especially in terms of pain reduction at 1 to 3 months post-treatment.

These short-term results are clinically relevant for patients with mild to moderate CTS who may not yet be candidates for surgery or who prefer to explore non-invasive treatments. Manual therapy offers a viable option for managing symptoms in the short term, with the potential to delay the need for surgery. While surgery remains the treatment of choice for long-term symptom resolution, the short-term efficacy of manual therapy adds value to a more tailored approach, allowing clinicians to offer patients individualized care based on their symptom severity and treatment preferences.

For patients with mild to moderate CTS, manual therapy offers a viable non-invasive option, especially for those seeking short-term relief. It is a cost-effective and low-risk intervention that can lead to meaningful improvements in pain and hand function within the first few months of treatment. However, for patients with severe CTS or those requiring long-term resolution of symptoms, surgery may be the preferred option due to its ability to provide more durable improvements in hand function and symptom severity. This review and meta-analysis demonstrate that both manual therapy and surgery are effective treatments for CTS, with each modality offering distinct advantages depending on the outcome and follow-up period. Manual therapy provides superior short-term benefits, particularly in reducing pain and improving hand function, while surgery offers greater long-term relief of symptom severity and hand function recovery. The meta-analyses conducted for pain demonstrate that manual therapy yields significant short-term improvements, particularly for pain. The overall moderate effect sizes suggest that manual therapy may offer a viable alternative to surgery for patients seeking non-invasive treatment options. Future research should aim to standardize manual therapy protocols, ensure longer follow-up periods, and address the limitations related to performance bias and treatment variability to further elucidate the comparative effectiveness of these interventions. These findings support the importance of individualized treatment approaches, with the choice of intervention guided by the patient’s clinical presentation, functional needs, and long-term goals.

### Clinical Practice Implications

The findings of this review suggest that both manual therapy and surgery are effective for treating Carpal Tunnel Syndrome (CTS), but each has specific advantages depending on the patient’s needs and severity of the condition. Manual therapy offers non-invasive, short-term relief of pain and improved hand function, making it ideal for patients with mild to moderate symptoms or those seeking to avoid surgery. It can be a first-line option for symptom management and may be used alongside other conservative treatments, like splinting and exercises.

Surgery, on the other hand, is more suitable for patients with severe or long-standing CTS who require long-term symptom relief. It provides durable improvements, particularly in hand function and symptom severity, and is recommended when conservative methods fail.

Clinicians should use a personalized approach when choosing between treatments, considering patient preferences, symptom severity, and long-term goals. Both interventions require follow-up, but manual therapy may need ongoing management, while surgery offers more permanent solutions. Shared decision making is key, and patients should be well informed about the benefits, risks, and expected outcomes of each treatment option.

## 5. Conclusions

This study highlights the significant short-term benefits of manual therapy in reducing pain for patients with Carpal Tunnel Syndrome (CTS), while confirming that surgery provides superior long-term improvements in hand function and symptom severity. The findings underscore the value of manual therapy as an effective, non-invasive option for patients seeking immediate relief, especially those with mild to moderate CTS or those looking to delay or avoid surgery. This study adds to the body of evidence supporting a tailored, patient-centered approach to CTS treatment, allowing for individualized decision making based on symptom severity, patient preferences, and treatment goals.

## Figures and Tables

**Table 1:** Participant characteristics.

Variables	Total Participants	Manual Therapy	Surgical Treatment
Number of people (%)	533 (100%)	268 (50.3%)	265 (49.7%)
Female (%)	479 (89.9%)	243 (90.7%)	236 (89.1%)
Male (%)	54 (10.1%)	25 (9.3%)	29 (10.9%)
Mean (SD)	Min	Max	Mean (SD)
Age	47.74 (1.71)	46	51
Years with pain	3.31 (0.3)	2.8	3.9
Unilateral CTS—Right	9.38 (2.62)	6	15
Unilateral CTS—Left	4.25 (2.1)	2	8
Bilateral symptoms	37.7 (4.57)	31	47
Home-based work	22.1 (9.43)	6	35
Office-based work	26.13 (2.1)	24	30
Mild CTS	13.88 (3.1)	10	18
Moderate CTS	20.13 (2.95)	16	24
Severe CTS	18 (2.93)	14	23
Pain intensity (NPRS 0–10)	5.18 (0.98)	4.2	6.8
BCTQ-FSS (1–5)	2.33 (0.14)	2.1	2.53
BCTQ-SSS (1–5)	2.68 (0.20)	2.5	3.01

Legend: BCTQ-FSS: Boston Carpal Tunnel Questionnaire—Functional Status Scale; BCTQ-SSS: Boston Carpal Tunnel Questionnaire—Symptom Severity Scale; CTS: Carpal Tunnel Syndrome; NPRS: Numeric Pain Rating Scale; SD: standard deviation.

**Table 2:** Summary of included studies.

Authors	Population	Intervention	Control	Follow-Up	Outcome: Evaluation Tool
Jarvik et al. 2009 [49]	116 patients positive for electrodiagnostic tests:	NSAIDs	Traditional or endoscopic decompression surgery.	6 and 12 months	Included in the review:
	Intervention group, n = 57.	Night wrist splint			Hand function: BCTQ-FSS.
	Control group, n = 59.	One visit per week for six weeks with hand exercises, ligament stretching, tendon gliding, and educational pamphlets provided at the first visit.			Symptom severity: BCTQ-SSS.
		If no improvement, 12 sessions of focused ultrasound therapy (2–4 per week for 6 weeks, 15 min each in pulsed mode 1:4, 1 MHz, 1.0 W/cm²).			Pain intensity: NPRS.
					Quality of life: SF-36.
					Excluded: Work days lost and reduced activity.
Fernández D. L. P. et al. 2015 [50]	120 women positive for clinical and electrodiagnostic tests:	One weekly treatment for three weeks of the following:	Traditional or endoscopic decompression surgery.	1, 3, 6, and 12 months	Included in the review:
	Intervention group, n = 60.	Soft tissue mobilization, tendon/nerve gliding exercises, and manual therapy techniques at possible median nerve entrapment sites.	Same education session as the intervention group.		Pain intensity: NPRS.
	Control group, n = 60.	Lateral gliding of the cervical spine and related tendon/nerve gliding.			Hand function: BCTQ-FSS.
		Educational session for tendon/nerve gliding exercises to perform at home during follow-up.			Symptom severity: BCTQ-SSS.
					Excluded: Self-perception of improvement (GROC scale).
Fernández D. L. P. et al. 2017 [51]	100 women positive for clinical and electrodiagnostic tests:	One weekly treatment for three weeks of the following:	Traditional or endoscopic decompression surgery.	3, 6, 9, and 12 months	Included in the review:
	Intervention group, n = 50.	Soft tissue mobilization, tendon/nerve gliding exercises, and manual therapy techniques at possible median nerve entrapment sites.	Same education session as the intervention group.		Pain pressure threshold.
	Control group, n = 50.	Upper-limb tendon/nerve gliding: two sets of 5 min with 1 min pause.			Pain intensity: NPRS.
		Educational session for tendon/nerve gliding exercises to perform at home during follow-up.			Thermal pain threshold.
Fernández D. L. P. et al. 2017 [53]	100 women positive for clinical and electrodiagnostic tests:	One weekly treatment for three weeks of the following:	Traditional or endoscopic decompression surgery.	1, 3, 6, and 12 months	Included in the review:
	Intervention group, n = 50.	Lateral gliding of the cervical spine and posterior–anterior pressure applied to the mid-cervical spine.	Same education session as the intervention group.		Hand function: BCTQ-FSS.
	Control group, n = 50.	Soft tissue intervention at potential median nerve entrapment sites.			Symptom severity: BCTQ-SSS.
		Cervical stretching exercises.			Excluded: Cervical range of motion (CROM device) and pinch grip strength (pinch dynamometer).
		Educational session for tendon/nerve gliding exercises to perform at home during follow-up.			
Fernández D. L. P. et al. 2020 [52]	120 women positive for clinical and electrodiagnostic tests:	One weekly treatment for three weeks of the following:	Traditional or endoscopic decompression surgery.	1 year and 4 years	Included in the review:
	Intervention group n = 60	Soft tissue mobilization, tendon/nerve gliding exercises, and manual therapy techniques at possible median nerve entrapment sites.	Same education session as the intervention group.		Pain intensity: NPRS.
	Control group n = 60	Lateral gliding of the cervical spine and related tendon/nerve gliding.			Hand function: BCTQ-FSS.
		Educational session for tendon/nerve gliding exercises to perform at home during follow-up.			Symptom severity: BCTQ-SSS.
					Excluded: Self-perception of improvement (GROC scale).

Legend: **BCTQ-FSS**: Boston Carpal Tunnel Questionnaire—Functional Status Scale; **BCTQ-SSS**: Boston Carpal Tunnel Questionnaire—Symptom Severity Scale; **CROM**: Cervical Range of Motion, **GROC**: Global Rating of Change Scale; **NPRS**: Numeric Pain Rating Scale; **NSAIDs**: Non-Steroidal Anti-Inflammatory Drugs; **SF-36**: Short Form-36 Health Survey.

## Data Availability

No new data were created or analyzed in this study.
